# Autonomic Nervous System Activity and Dental Anxiety in the Northern Finland Birth Cohort (NFBC1966) Population

**DOI:** 10.3390/dj12030081

**Published:** 2024-03-21

**Authors:** Atte Somero, Auli Suominen, Vesa Pohjola, Mika Ogawa, Kirsi Sipilä, Niklas Kakko, Mikko Tulppo, Satu Lahti

**Affiliations:** 1 Department of Community Dentistry, University of Turku, 20014 Turku, Finland; atte.h.somero@utu.fi (A.S.); auli.suominen@utu.fi (A.S.); vesa.pohjola@utu.fi (V.P.); mika.ogawa@utu.fi (M.O.); 2 Research Unit of Population Health, Faculty of Medicine, University of Oulu, 90014 Oulu, Finland; kirsi.sipila@oulu.fi (K.S.); niklas.kakko@student.oulu.fi (N.K.); 3 Medical Research Center Oulu, Oulu University Hospital, University of Oulu, 90014 Oulu, Finland; mikko.tulppo@oulu.fi; 4 Research Unit of Biomedicine and Internal Medicine, University of Oulu, 90014 Oulu, Finland

**Keywords:** dental anxiety, autonomic nervous system, comorbidity, NFBC1966

## Abstract

Autonomic nervous system (ANS) activity may be associated with dental anxiety; however, no studies exist on the topic. The aim of this study was to assess if ANS activity and dental anxiety are associated. Data were collected as part of a Northern Finland Birth Cohort Study, NFBC1966, at the age of 46 years with eligible data on 1905 participants. Dental anxiety was measured using the Modified Dental Anxiety Scale (MDAS) categorized as follows: 19–25 = high, 10–18 = moderate, 5–9 = no to low dental anxiety. Heart rate variability (HRV) data were collected using an HR monitor and a standard lead-II electrocardiogram (ECG). Baroreflex sensitivity (BRS) was calculated from continuous ECG and blood pressure signals. Participants were categorized into three groups as follows: Low = the lowest 20th percentile, Mid = medium 21–79th percentile, and High = the highest 20th percentile according to their ANS variables. The associations between the MDAS and ANS activity parameters were evaluated using unordered multinomial logit models adjusted for comorbidities, β-blockers, BMI, smoking, and education. High heart rate, reduced HRV, low cardiac vagal modulation, and poor BRS were associated with moderate dental anxiety, and high cardiac vagal modulation and good BRS were associated with no to low dental anxiety. Poorer ANS activity might predispose some to dental anxiety, and better ANS activity might protect from dental anxiety.

## 1. Introduction

Every third adult Finn suffers from dental anxiety and fear [1]. Thus, dental anxiety is very common, and dentists meet these patients daily. Dental anxiety levels differ according to age, gender, and regular attendance to oral health care [1,2,3,4]. Women and younger people report dental anxiety more often than older people and men do [1,2,3,4]. Dental anxiety has been shown to lead to irregular dental attendance [3]. Those with dental anxiety also have poorer oral health [4].

Dental anxiety contains physical and psychological aspects. Dental anxiety is associated with several psychological symptoms and disorders, such as depression and general anxiety, and phobias [5,6,7]. However, a recent study showed that temperament traits erased the effect of depressive and general anxiety symptoms [8], suggesting that stable individual characteristics may also play an important role in the development of dental anxiety besides a person’s own or vicariously learned negative experiences [9].

Common physiological changes associated with dental anxiety are, for example, increased heart rate, excessive sweating, and nervousness [10]. Changes are likely to be due to altered autonomic nervous system (ANS) activity. In the Diagnostic and Statistical Manual of Mental Disorders, dental anxiety has been categorized under specific phobias in the sub-category Blood-Injection-Injury (BII) phobia, which is characterized by a strong vasovagal response [11]. BII has in turn been associated with anxiety sensitivity, which is the fear of arousal-related sensations due to the belief that such sensations will have negative implications [12,13]. Though the diagnostic categorization of dental anxiety under BII has been justifiably questioned [14], physiological arousal, beyond that which is vasovagal, and the fear of this arousal may be one important etiological factor of dental anxiety. In addition, excessive stress caused by anxiety can rapidly activate the parasympathetic nervous system, which is triggered by pain or other stimuli, thereby causing bradycardia or sometimes syncope. This vasovagal reflex is reported to be the most frequent emergency in dental practice [15,16]. Thus, understanding dental anxiety and ANS activity and its response is essential for dental clinicians.

There are different measures of ANS activity. Heart rate variability (HRV) is the variation between heartbeats and it is a common measure for assessing the state of ANS. HRV analysis can indicate how sensitive the subject’s autonomic state is. HRV frequency includes two values as follows: low frequency (LF) and high frequency (HF). The LF value is used to assess parasympathetic and sympathetic ANS activity, while the HF one is a more specific value as it measures mainly parasympathetic activity. The LF/HF ratio can be calculated from the frequency values, and it measures sympathovagal balance. The baroreceptors are located in carotid sinuses, and they modulate blood pressure and heart rate. Baroreflex sensitivity (BRS) measures both vagal and sympathetic activity response to stressors.

Though ANS activity is likely to be associated with dental anxiety, we could not identify studies reporting whether ANS activity measured in a non-dental context is associated with dental anxiety in the general population. Thus, this study aimed to assess if ANS activity and dental anxiety are associated. Our hypothesis was that those with high ANS activity, especially vasovagal, are more likely to have dental anxiety.

## 2. Materials and Methods

This study utilizes data that was collected in a part of a Northern Finland Birth Cohort, NFBC1966, in two northern provinces of Oulu and Lapland (n = 12,231) [17,18]. Data used in this research were collected during the latest follow-up at the age of 46 years (years 2012–2014), containing a subpopulation living within 100 km from the city of Oulu examined in a field study. There were 3150 participants alive and with known addresses. The total number of recruited individuals was 1964; 62.3% of those who were invited to participate in the study. Of those, two participants refused the use of their data, and the final 1962 participants fulfilled the inclusion criteria of having sufficient data on dental anxiety and ANS activity and were included in the study.

Data were acquired using questionnaires and health examinations. The study followed the principles of the Declaration of Helsinki. The Ethics Committee of the Northern Ostrobothnia Hospital District approved the research (74/2011). Participants’ rights were protected by an appropriate Institutional Review Board. Written informed consent was obtained from all participants [17,18].

The participant sat on a chair for instrumentation and the review of the protocol. A heart rate (HR) monitor (RS800CX, Polar Electro Oy, Kempele, Finland) was used to record R-R intervals (RRi) with an accuracy of 1 ms. In about half of the participants (Oulu laboratory unit only), spontaneous BRS was also assessed. Standard lead-II ECG (Cardiolife, Nihon Kohden, Tokyo, Japan), breathing frequency (MLT415/D, Nasal Temperature Probe, ADInstruments, Bella Vista, New South Wales, Australia), and blood pressure (BP) by finger plethysmography (Nexfin, BMEYE Medical Systems, Amsterdam, The Netherlands) were recorded during the protocol with a sampling frequency of 1000 Hz (PowerLab 8/35, ADInstruments). The examination time was 6 min, with the first 3 min seated and the last 3 min standing. Examinations were preceded by at least a 1 min stabilization period. The first 150 s while seated and the last 150 s in a standing position were used in the HRV analyses.

The Rri data were edited based on visual inspection (Hearts 1.2, University of Oulu, Oulu, Finland). Artefacts and ectopic beats were removed and replaced by the local average. However, sequences with ≥10 consecutive beats of noise or ectopic beats were deleted. The Rri series with ≥80% accepted data were included in analyses. Mean HR, root mean square of successive differences in Rri (rMSSD, ms), spectral power densities (fast Fourier transform, length 512 beats) at the low-frequency (LF, 0.04–0.15 Hz, ms^2^) and high-frequency (HF, 0.15–0.40 Hz, ms^2^) components of HRV and their ratio (LF/HF) were analyzed.

Baroreflex sensitivity was calculated through continuous ECG, blood pressure, and respiration signal data. Calculations were carried out by Matlab-based software (Biosignal processing team, University of Oulu, Finland). SBP and RR interval data were extracted from the recordings and were further used. Artefacts and ectopic beats were replaced using linear interpolation (<5% for accepted recording) and, thereafter, resampled at 2 Hz. Very low-frequency components (<0.04 Hz) were removed using the Savitzky–Golay method. A fast Fourier transform (Welch method, segments of 128 samples with 50% overlap, length 1024 samples) was performed to analyze the LF power of Rri and systolic BP oscillations (ms^2^, mmHg^2^) for subsequent analysis of BRS by the alpha method if sufficient coherence (≥0.5) between LF oscillations in Rri and systolic BP was verified. Participants were further categorized into three categories as follows: Low = the lowest 20th percentile, Mid = medium 21–79th percentile, High = the highest 20th percentile, according to their HRV and Baroreflex variables.

Dental anxiety was measured two hours before the clinical health examination using the Modified Dental Anxiety Scale (MDAS). The MDAS is a valid and reliable five-item questionnaire for self-estimating dental anxiety [19,20]. The five questions in the MDAS questionnaire are as follows: 1. If you went to your dentist for treatment tomorrow, how would you feel? 2. If you were sitting in the waiting room (waiting for treatment), how would you feel? 3. If you were about to have a tooth drilled, how would you feel? 4. If you were about to have your teeth scaled and polished, how would you feel? 5. If you were about to have a local anesthetic injection in your gum, about an upper back tooth, how would you feel? Response options given are as follows: Not anxious, Slightly anxious, Fairly anxious, Very anxious, and Extremely anxious. Scoring of the answers were 1 (not anxious), 2 (slightly anxious), 3 (fairly anxious), 4 (very anxious), and 5 (extremely anxious).

A total sum of the MDAS score varying between 5 and 25 was calculated. Participants were further divided into three categories according to their MDAS score (19–25 = high dental anxiety, 10–18 = moderate dental anxiety, 5–9 = no to low dental anxiety).

Background variables included those that have been systematically associated with dental anxiety [1,21] and ANS activity [22,23,24,25], which are sex assigned at birth; comorbidities (including diabetes mellitus, cardiovascular diseases, and fibromyalgia); use of beta blockers; BMI; smoking categorized as former, current, or nonsmoker; and educational level categorized as elementary school, high school, or university level.

The percentiles of each cardiovascular ANS activity parameter were calculated by sex. The three-class categorization (Low, Mid, High) for each participant was performed based on parameter specific percentiles.

Descriptive and bivariate statistical analyses were conducted as follows: Categorical variables were described with frequencies and percentages. The means and standard deviations were calculated for continuous variables. The normality of continuous variables was confirmed visually with graphs. The associations between background variables and categorical MDAS were analyzed through the Chi-square test. The associations between continuous ANS activity parameters and the categorical MDAS were analyzed with one-way ANOVA. If the normality assumption failed, the Kolmogorov–Smirnov test was used.

Multivariable statistical analyses were conducted as follows: The associations between the MDAS and cardiovascular ANS activity parameters were evaluated using unordered multinomial logit models. The models were adjusted for selected background variables. The backward selection criterion was used for covariates and their interaction terms with the limit of *p*-value < 0.05. Separate analyses were conducted for every cardiovascular ANS activity parameter as independent variables. The dependent variable was the categorized MDAS. First, separate analyses were executed for each continuous cardiovascular ANS activity parameter. Secondly, the analyses were repeated for categorized cardiovascular ANS activity parameters (medium percentiles as reference). Odds ratios (ORs) and 95% confidence intervals (95% CIs) were calculated for both MDAS categories (no to low dental anxiety as reference).

The statistical software SPSS (IBM Corp. IBM SPSS Statistics for Windows, Version 25.0.; IBM Corp., Armonk, NY, USA, 2022) and SAS (Statistical Analysis Software 9.4, SAS Institute Inc., Cary, NC, USA) was used for statistical analysis. *p*-value < 0.05 was considered to be statistically significant.

## 3. Results

Of the participants, 53.4% (n = 1017) were females and 46.6% (n = 888) were males. The mean MDAS score was 9.3 (SD = 4.0) for all participants, 10.1 (SD = 4.4) for females, and 8.2 (SD = 3.2) for males (*p* < 0.001). Of the participants, 4.6% had high dental anxiety, 32.2% moderate dental anxiety, and 63.2% no to low dental anxiety. Females reported more often dental anxiety 6.9% (high), 37.9% (moderate) and 55.3% (no to low) than males 1.9% (high), 25.8% (moderate) and 72.3% (no to low), (*p* < 0.001).

Of the study participants, 6.1% had elementary level school education, 37.5% elementary school level education, and 56.4% had university level education, with females having higher education (5.8%, 29.8%, and 64.4%) than males (6.5%, 46.3%, 47.2%) respectively (*p* < 0.001). Of the participants, 21.3% were current smokers, 23.8% former smokers, and 54.9% nonsmokers, with females being regular smokers less often (19.8%, 20.6%, 59.6%) than males (23.1%, 27.5%, 39.4%) (*p* ≤ 0.001), respectively. The differences between sexes were statistically significant in educational level and smoking history (*p* =< 0.001). The mean BMI was 26.71 (SD = 4.6). Beta-blockers were used by 6.8% of participants and (22.5%) had comorbidities (cardiovascular diseases, diabetes mellitus, or fibromyalgia).

Table 1 shows the mean levels of cardiovascular ANS activity and BMI and the prevalence of other covariates by dental anxiety levels. Those with high dental anxiety reported more often the use of beta blockers and were more often current smokers than those with moderate or low dental anxiety. High dental anxiety was also positively associated with the comorbidity of diabetes, cardiovascular diseases, and fibromyalgia, indicating more health problems in those with dental anxiety. Those with no to low dental anxiety had a lower heart rate, higher sympathetic activity (LF power, LF/HF ratio, as well as systolic and diastolic BP) and better baroreflex when seated. When standing, they had a lower heart rate, higher HRV, and higher sympathetic activity (LF/HF ratio, systolic BP).

In the final model, when adjusted for BMI, sex, and smoking, only HR was statistically significantly associated with dental anxiety. Those with moderate dental anxiety had a higher HR than those with no or low dental anxiety when both seated (OR = 1.013, 95% CI = 1.004–1.030, *p* = 0.004) and standing (OR = 1.009, 95% CI = 1.001–1.016, *p* = 0.032).

**Figure 1:**
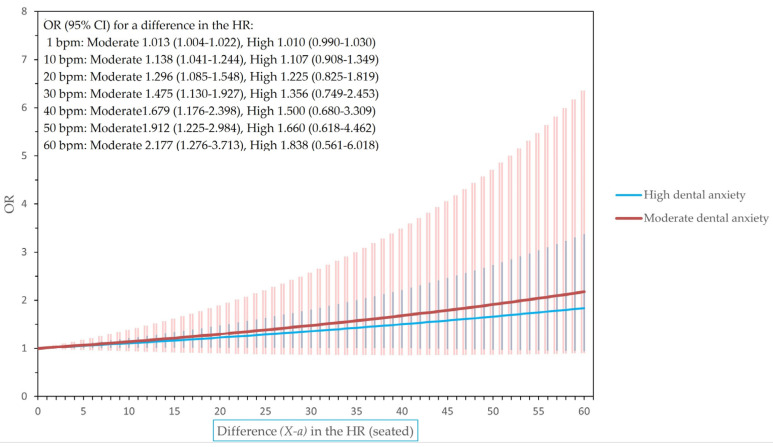
The odds ratios (ORs) of having high or moderate dental anxiety for a difference in heart rate (HR) when seated (i.e., odds of having X HR in relation to the corresponding odds of having X-*a* HR, where X may be any HR level and *a* any chosen value (0–60) in the level of HR).

**Figure 2:**
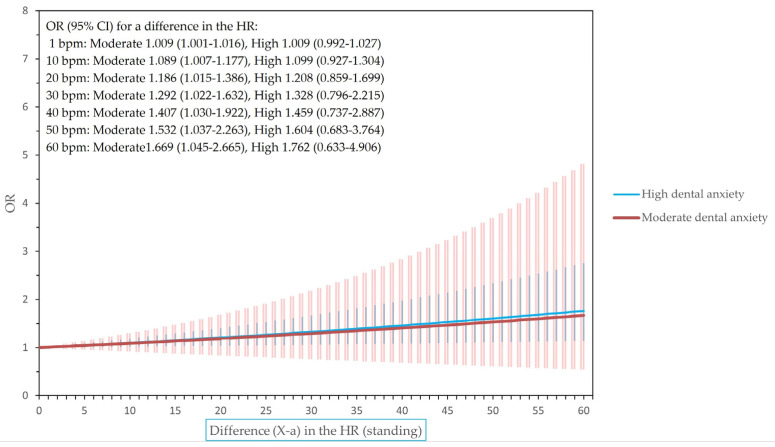
The odds ratios (ORs) of having high or moderate dental anxiety for a difference in heart rate (HR) when standing (i.e., odds of having X HR in relation to the corresponding odds of having X-*a* HR, where X may be any HR level and *a* any chosen value (0–60) in the level of HR).

**Figure 3:**
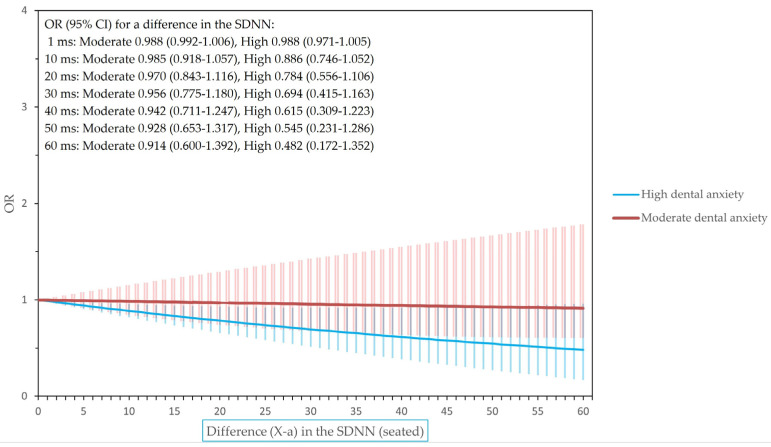
The odds ratios (ORs) of having high or moderate dental anxiety for a difference in SDNN when seated (i.e., odds of having X SDNN in relation to the corresponding odds of having X-*a* SDNN, where X may be any SDNN level and *a* any chosen value (0–60) in the level of SDNN).

Figure 1, Figure 2 and Figure 3 show the odds ratios for differences in the heart rate and heart rate variability for final models adjusted for BMI, sex, and smoking.

Table 2 shows the prevalence of different levels of dental anxiety by ANS activity standardized by sex and the unordered multinomial logit final models adjusted for BMI and smoking. Compared to those 60% with a medium heart rate, those 20% with the highest heart rate when seated were more likely (OR = 1.50, 95% CI = 1.16–1.93) to have moderate than no to low dental anxiety. When comparing to those 60% medium HRV (SDNN), those 20% with smallest HRV when seated were more likely to have moderate than no to low dental anxiety (OR = 1.33, 95% CI = 1.01–1.73; while those with 20% highest HRV when standing were less likely to have moderate than no to low dental anxiety (OR = 0.74, 95% CI = 0.16–0.97).

Compared to those 60% with medium cardiac vagal modulation (rMSDD), those 20% with the lowest rMSDD when seated were more likely (OR = 1.5, 95% CI = 1.2–2.0) to have moderate dental anxiety than no to low dental anxiety. When compared to those 60% with medium cardiac vagus nerve activity (HF), those 20% with the smallest HF when standing were more likely (OR = 1.4, 95% CI = 1.1–1.8) to have moderate than no to low dental anxiety and those 20% with the highest HF when standing were less likely (OR = 0.5, 95% CI = 0.2–0.9) to have high than no to low dental anxiety.

Compared to those 60% with medium baroreflex activity (LF), those 20% with the lowest LF when seated were more likely (OR = 1.5, 95% CI = 1.1–1.9) to have moderate than no to low dental anxiety. When compared to those 60% with medium baroreflex sensitivity (BRS), those 20% with the poorest BRS when seated were more likely (OR = 1.5, 95% CI = 1.1–1.9) to have moderate than no to low dental anxiety and those 20% with the best BRS were less likely (OR = 0.4, 95%CI = 0.2–0.9) to have high than no to low dental anxiety.

## 4. Discussion

Of the cardiovascular ANS activity parameters, high heart rate, reduced heart rate variability, low cardiac vagal modulation, and poor baroreflex sensitivity seemed to predispose some to moderate dental anxiety when adjusted for important covariates. In addition, high cardiac vagal modulation and good baroreflex seemed to protect from high dental anxiety. However, the direction of the causality needs to be confirmed in a longitudinal set-up.

A novel finding was also that those with comorbidities or using beta blockers had higher levels of dental anxiety on a population level. This suggests that individuals who have severe dental anxiety are also more likely to have other diseases and medication. The previous finding that smokers reported higher levels of dental anxiety was also confirmed [21]. Besides regular smoking [21], dental anxiety has been related to high body mass index [26] and poor physical well-being [27].

Of the comorbidities, fibromyalgia is associated with higher pain sensitivity and reduced vagal activity, as indicated by reduced heart rate variability, as has been reported [25]. A previous study using the same cohort also noted an association between dental anxiety and pain sensitivity [28]. Coronary artery disease and diabetes are also associated with oral health [29,30,31]. Beta-blockers are widely used for treating coronary artery diseases [32]. As dental anxiety leads to avoidance of treatment [3], which in turn leads to poor oral health [3,33], our findings may indicate that oral health influences systemic diseases and that dental anxiety may reinforce this relationship. However, in this study we did not analyze each comorbidity separately as they were considered as confounders. Nor was the clinical oral health condition analyzed. Thus, further studies are needed.

Previous studies indicate [34] that patients with higher dental anxiety show a higher increase in heart rate than those with modest dental anxiety. An elevated heart rate can be seen before and during the treatment [35,36]. This suggests that ANS activity of dentally anxious patients might differ from those of non-anxious ones. However, we could not identify studies reporting whether ANS activity measured in a non-dental context is associated with dental anxiety in the general population.

A meta-analysis by Chalmer et al. [37] reports that anxiety disorders such as panic disorder, post-traumatic stress disorder, and generalized anxiety disorder are associated with lower heart rate variability (especially HF). Panic disorder is also reported to be independently associated with cardiovascular disease [38]. As mechanisms for the association between anxiety and cardiovascular disease, it has been considered that anxiety may lead to higher threat-related attentional bias, increased activation of the stress response, chronically low parasympathetic activity, impaired cholinergic anti-inflammatory reflexes, and increased risk of diseases, including diabetes [37,39,40,41]. The results of the present study are consistent with these findings, and anxiety about dental treatment, as well as other anxiety disorders, may be a factor in reducing vagal activity and harming overall health.

The strengths of our study include a large representative population-based cohort sample; the use of reliable and valid measures for dental anxiety, ANS activity, and confounders; and how ANS activity was measured in non-dental context. The study also has limitations. Dental fear was measured in conjunction with oral examination. The lower percentage of males with high dental anxiety (2%) than in the national survey (4%) [1] might indicate that males with high dental anxiety avoided participation in the study due to oral examination [29]. This might have affected the results so that differences between high dental anxiety groups were not observed. In addition, the study group was limited to one age group only. Use of the LF/HF ratio has also had its downsides according to some studies [42,43]. As this study was nested in a large cohort study, no power analysis specific to this study was performed.

This study has some practical implications. A feeling of control is crucial for those with dental anxiety [44]. For some, laying down in dental chair causes lack of control. On the other hand, when treated in a half-sitting position, low baroreflex can affect blood pressure, which in turn can increase the feeling of dizziness and complicate the situation.

## 5. Conclusions

Patients with poorer ANS activity might be more vulnerable to dental anxiety. Poorer ANS activity can also complicate treatment for those with dental anxiety. Also, clinicians should consider the common risk of dental anxiety with smoking and comorbidities. Thus, understanding the role of ANS activity is important for dental practitioners. However, due to the cross-sectional design, more research is needed to assess the causal direction.

## Figures and Tables

**Table 1:** Cardiovascular ANS ^1^ activity, BMI ^2^, comorbidities (diabetes mellitus, cardiovascular diseases, fibromyalgia), use of beta-blockers, educational level, and smoking in participants with high, moderate, and no to low dental anxiety.

	Dental Anxiety			*p*-Level	Adjusted *p*-Level		
	High n = 87	Moderate n = 614	No to Low n = 1204	All	All	High vs. Low	Moderate vs. No to Low
Male (%)	1.9	25.8	72.3	**<0.001**	n/a	n/a	n/a
Female (%)	6.9	37.9	55.3				
BMI	28 ± 7	27 ± 5	27 ± 4	0.089	n/a	n/a	n/a
Comorbidity (%)	29.9	26.2	20.1	**0.003**	n/a	n/a	n/a
β-blockers (%)	12.6	6.4	4.3	**0.001**	n/a	n/a	n/a
**Education (%)**							
Elementary	8.1	6.7	5.7	0.823	n/a	n/a	n/a
High	39.1	37.0	37.6		n/a	n/a	n/a
University	52.9	56.4	56.6		n/a	n/a	n/a
**Smoking (%)**							
Current	33.3	24.6	18.8	**0.002**	n/a	n/a	n/a
Former	24.1	22.5	24.5		n/a	n/a	n/a
Nonsmoker	42.5	52.9	56.7		n/a	n/a	n/a
**ANS seated**							
HR ^3^, bpm	77 ± 12	77 ± 12	75 ± 11	**0.005**	**0.015**	0.315	**0.004**
SDNN ^4^, ms	30 ± 14	32 ± 16	33 ± 14	0.082	0.374	0.166	0.674
rMSSD ^5^, ms	21 ± 16	21 ± 13	21 ± 12	0.619	0.700	0.876	0.399
HF ^6^, ln ms^2^	4.8 ± 1.3	4.8 ± 1.3	4.9 ± 1.3	0.708	0.426	0.550	0.211
LF ^7^, ln ms^2^	5.2 ± 1.1	5.4 ± 1.1	5.2 ± 1.0	**0.016**	0.271	0.127	0.428
LF/HF	2.1 ± 2.1	2.8 ± 3.2	2.9 ± 3.3	**0.037**	0.166	0.144	0.300
SBP ^8^, mmHg	112 ± 16	116 ± 16	117 ± 15	**0.017**	0.186	0.311	0.185
DBP ^9^, mmHg	68 ± 9	69 ± 9	70 ± 10	**0.032**	0.672	0.482	0.681
LF-SBP, mmHg^2^	7.1 ± 8.3	7.7 ± 7.5	7.6 ± 8.0	0.310	0.770	0.778	0.543
BRS ^10^, ms/mmHg	6.8 ± 3.7	7.0 ± 3.8	7.5 ± 4.1	**0.039**	0.195	0.355	0.085
**ANS standing**							
HR, bpm	88 ± 13	87 ± 13	86 ± 13	**0.021**	0.078	0.278	**0.032**
SDNN, ms	25 ± 13	27 ± 13	29 ± 13	**<0.001**	0.072	0.119	0.059
rMSSD, ms	13 ± 11	13 ± 9	13 ± 9	0.211	0.768	0.958	0.470
HF, ln ms^2^	3.8 ± 1.4	3.9 ± 1.4	3.9 ± 1.4	0.324	0.557	0.764	0.147
LF, ln ms^2^	3.8 ± 1.4	3.9 ± 1.4	3.9 ± 1.4	0.324	0.139	0.134	0.122
LF/HF	4.1 ± 4.0	4.7 ± 4.2	5.2 ± 5.1	**0.044**	0.589	0.326	0.657
SBP, mmHg	112 ± 16	115 ± 16	116 ± 15	**0.033**	0.716	0.462	0.827
DBP, mmHg	70 ± 10	71 ± 9	72 ± 9	0.112	0.696	0.540	0.488
LF-SBP, mmHg^2^	9.9 ± 8.3	10.9 ± 11.3	11.8 ± 12.0	0.176	0.936	0.717	0.934
BRS, ms/mmHg	4.6 ± 2.9	4.9 ± 3.0	5.1 ± 3.0	0.074	0.601	0.394	0.508

^1^ ANS: autonomic nervous system, ^2^ BMI: body mass index, ^3^ HR: heart rate, ^4^ SDNN: standard deviation of normal-to-normal R-R interval, ^5^ rMSSD: root mean square of successive differences in R-R intervals, ^6^ HF: high-frequency power of R-R interval oscillations, ^7^ LF: low-frequency power of R-R intervals oscillations, ^8^ SBP: systolic blood pressure, ^9^ DBP: diastolic blood pressure, ^10^ BRS: baroreflex sensitivity.

**Table 2:** Prevalence of different levels of dental anxiety according to the level of ANS ^1^ activity, i.e., the lowest and highest 20% and the mid group (as a reference group) standardized according to sex at birth.

	Dental Anxiety				Adjusted *p*-Level	
	High n = 87	Moderate n = 614	No to Low n = 1204	*p*-Level	Moderate vs. No to Low	High vs. No to Low
**ANS seated**	%	%	%			
HR ^2^, low	19.8	18.2	20.8	**0.018**	0.732	0.696
HR, mid	57.0	57.5	61.6		reference	reference
HR, high	23.3	24.3	17.7		**0.002**	0.271
SDNN ^3^, low	23.3	20.6	16.2	0.054	**0.039**	0.329
SDNN, mid	63.9	59.5	62.9		reference	reference
SDNN, high	12.8	19.9	20.9		0.951	0.147
rMSSD ^4^, low	20.0	24.3	17.5	**0.011**	**0.001**	0.977
rMSSD, mid	64.7	56.4	61.9		reference	reference
rMSSD, high	15.3	19.3	20.6		0.941	0.403
HF ^5^, low	21.2	23.1	17.6	0.118	**0.006**	0.894
HF, mid	67.1	56.5	62.1		reference	reference
HF, high	11.8	20.3	20.4		0.756	0.132
LF ^6^, low	25.6	22.9	17.5	**0.004**	**0.004**	0.300
LF, mid	64.0	55.6	62.7		reference	reference
LF, high	10.5	21.5	19.9		0.111	0.080
LF/HF, low	13.9	20.5	20.1	**0.017**	0.604	0.104
LF/HF, mid	73.3	58.1	60.1		reference	reference
LF/HF, high	12.8	21.4	19.8		0.253	0.060
BRS ^7^, low	22.6	23.7	17.9	**0.006**	**0.003**	0.835
BRS, mid	69.1	56.6	61.2		reference	reference
BRS, high	8.3	19.7	20.9		0.886	**0.022**
**ANS standing**	%	%	%			
HR, low	23.3	18.2	20.7	0.211	0.267	0.380
HR, mid	55.8	59.0	60.8		reference	reference
HR, high	20.9	22.8	18.6		0.069	0.543
SDNN, low	24.7	19.7	15.4	**0.004**	0.101	0.182
SDNN, mid	61.2	63.0	61.7		reference	reference
SDNN, high	14.1	17.3	22.9		**0.027**	0.259
rMSSD, low	16.7	22.3	18.7	0.165	0.118	0.2164
rMSSD, mid	69.4	58.1	60.6		reference	reference
rMSSD, high	14.1	19.6	20.7		0.816	0.163
HF, low	18.8	22.9	18.3	0.376	**0.019**	0.402
HF, mid	71.8	56.3	61.3		reference	reference
HF, high	9.4	20.8	20.4		0.473	**0.030**
LF, low	26.7	21.1	18.7	0.194	0.471	0.298
LF, mid	58.1	60.1	60.1		reference	reference
LF, high	15.1	18.3	21.2		0.305	0.380
LF/HF, low	25.6	19.7	19.7	**0.011**	0.940	0.330
LF/HF, mid	61.3	59.2	60.5		reference	reference
LF/HF, high	12.8	21.1	19.8		0.630	0.177
BRS, low	23.5	20.1	19.5	0.580	0.760	0.735
BRS, mid	62.4	60.8	59.7		reference	reference
BRS, high	14.1	19.1	20.8		0.510	0.198

^1^ ANS: autonomic nervous system, ^2^ HR: heart rate, ^3^ SDNN: standard deviation of normal-to-normal R-R-interval, ^4^ rMSSD: root mean square of successive differences in R-R intervals, ^5^ HF: high-frequency power of R-R intervals oscillations, ^6^ LF: low-frequency power of R-R interval oscillations, ^7^ BRS: baroreflex sensitivity.

## Data Availability

The data that support the findings of this study are available at the NFBC for researchers who meet the criteria for accessing confidential data. Please contact the NFBC project center (nfbcprojectcenter@oulu.fi) and visit the cohort website for more information.
